# Supplementary material for: Role and mechanism of NCAPD3 in promoting malignant behaviors in gastric cancer
Source: Front Pharmacol. 2024 Apr 22;15:1341039. doi: 10.3389/fphar.2024.1341039 (PMC11070777; doi:10.3389/fphar.2024.1341039)
Supplement: Supplementary file 11 [file DataSheet2.ZIP › GSEA/Transcipt factor/my_analysis.Gsea.1599461814734/TCCCRNNRTGC_UNKNOWN.html]

Details for gene set TCCCRNNRTGC\_UNKNOWN[GSEA]

|  || Dataset | filtered\_dataset.sample\_info.cls#WT\_versus\_NCAPD3\_MUT |
| Phenotype | sample\_info.cls#WT\_versus\_NCAPD3\_MUT |
| Upregulated in class | WT |
| GeneSet | TCCCRNNRTGC\_UNKNOWN |
| Enrichment Score (ES) | 0.3618768 |
| Normalized Enrichment Score (NES) | 1.6219171 |
| Nominal p-value | 0.023569023 |
| FDR q-value | 0.5484255 |
| FWER p-Value | 1.0 |
Table: GSEA Results Summary

  

Fig 1: Enrichment plot: TCCCRNNRTGC\_UNKNOWN      
 Profile of the Running ES Score & Positions of GeneSet Members on the Rank Ordered List

  

| SYMBOL | TITLE | RANK IN GENE LIST | RANK METRIC SCORE | RUNNING ES | CORE ENRICHMENT || 1 | 2935 | GSPT1 | 5 | 1.156 | 0.0887 | Yes |
| 2 | 7743 | ZNF189 | 74 | 0.851 | 0.1077 | Yes |
| 3 | 4836 | NMT1 | 208 | 0.669 | 0.0653 | Yes |
| 4 | 54801 | HAUS6 | 219 | 0.660 | 0.1109 | Yes |
| 5 | 6426 | SRSF1 | 243 | 0.637 | 0.1452 | Yes |
| 6 | 55756 | INTS9 | 257 | 0.624 | 0.1857 | Yes |
| 7 | 84078 | KBTBD7 | 295 | 0.594 | 0.2065 | Yes |
| 8 | 79443 | FYCO1 | 313 | 0.586 | 0.2411 | Yes |
| 9 | 55869 | HDAC8 | 352 | 0.566 | 0.2590 | Yes |
| 10 | 5036 | PA2G4 | 365 | 0.557 | 0.2948 | Yes |
| 11 | 10426 | TUBGCP3 | 402 | 0.535 | 0.3116 | Yes |
| 12 | 29071 | C1GALT1C1 | 457 | 0.500 | 0.3126 | Yes |
| 13 | 25880 | TMEM186 | 462 | 0.498 | 0.3495 | Yes |
| 14 | 64324 | NSD1 | 534 | 0.455 | 0.3347 | Yes |
| 15 | 55252 | ASXL2 | 547 | 0.448 | 0.3619 | Yes |
| 16 | 1108 | CHD4 | 627 | 0.408 | 0.3376 | No |
| 17 | 23212 | RRS1 | 681 | 0.387 | 0.3303 | No |
| 18 | 6651 | SON | 716 | 0.365 | 0.3350 | No |
| 19 | 55122 | AKIRIN2 | 721 | 0.363 | 0.3611 | No |
| 20 | 79590 | MRPL24 | 800 | 0.320 | 0.3305 | No |
| 21 | 1984 | EIF5A | 846 | 0.259 | 0.3188 | No |
| 22 | 25901 | CCDC28A | 946 | -0.366 | 0.2767 | No |
| 23 | 7414 | VCL | 1308 | -0.712 | 0.0735 | No |
Table: GSEA details [plain text format]

  

Fig 2: TCCCRNNRTGC\_UNKNOWN      
 Blue-Pink O' Gram in the Space of the Analyzed GeneSet

  

Fig 3: TCCCRNNRTGC\_UNKNOWN: Random ES distribution      
 Gene set null distribution of ES for **TCCCRNNRTGC\_UNKNOWN**
